# Mycobacterium Avium Complex Genitourinary Infections: Case Report and Literature Review

**DOI:** 10.3390/idr13020045

**Published:** 2021-05-24

**Authors:** Sanu Rajendraprasad, Christopher Destache, David Quimby

**Affiliations:** 1 School of Medicine, Creighton University, Omaha, NE 68124, USA; SanuRajendraprasad@creighton.edu; 2 College of Pharmacy, Creighton University, Omaha, NE 68124, USA; chris.destache@creighton.edu

**Keywords:** nontuberculous mycobacteria, mycobacterium avium-intracellulare complex, urinary tract infections, genitourinary infections

## Abstract

Nontuberculous mycobacterial (NTM) genitourinary (GU) infections are relatively rare, and there is frequently a delay in diagnosis. Mycobacterium avium-intracellulare complex (MAC) cases seem to be less frequent than other NTM as a cause of these infections. In addition, there are no set treatment guidelines for these organisms in the GU tract. Given the limitations of data this review summarizes a case presentation of this infection and the literature available on the topic. Many different antimicrobial regimens and durations have been used in the published literature. While the infrequency of these infections suggests that there will not be randomized controlled trials to determine optimal therapy, our case suggests that a brief course of amikacin may play a useful role in those who cannot tolerate other antibiotics.

## 1. Introduction

In recent decades, the incidence and prevalence of nontuberculous mycobacteria (NTM) causing extrapulmonary infections have greatly increased, becoming a major worldwide public health problem [1,2]. Among numerous NTM species, the *Mycobacterium avium* complex (MAC) is the most common cause of infection in humans. MAC is recognized as a ubiquitous microorganism, with contaminated water and soil as established sources of infection [3]. MAC consists of multiple species, of which two of the most common are *M.avium* and *M.intracellulare* [4,5,6]. There are four main manifestations of MAC infection: (1.) HIV with disseminated disease; (2.) focal lymphadenitis also in predominantly HIV patients; (3.) pulmonary infection in those with structural lung disease; and (4.) skin and soft tissue infections [7,8,9,10]. Genitourinary tract infections secondary to MAC appear to be rare with questionable clinical relevance and no standardized treatment. Here we present a case of MAC-associated urinary tract infection in a woman with an extensive urological history and recurrent urinary tract infections.

## 2. Case Presentation

Patient is a 79-year-old woman with a complicated urologic history. Three years prior to her presentation in the infectious disease clinic, she had issues with urinary retention and frequent urinary tract infections, for which she would receive antibiotics. She required an indwelling urinary catheter for several months due to urinary retention, but voiding trials after initiation of tamsulosin were effective, and she was catheter-free for over 2 years prior to infectious disease clinic presentation. Recurrent infections were treated with methenamine and she had no symptoms of infection between November 2016 and June 2017. Starting in June 2017, she had more issues with urinary tract infection (UTI) symptoms, which improved with thrice-weekly vaginal estrogen cream and intermittent antibiotics. 

Beginning in January 2019, however, her symptoms became near-constant. Repeated urine cultures were positive for methicillin-sensitive *Staphylococcus aureus* (Table 1); she was found to have some kidney stones, which were removed in February of 2019. Unfortunately, despite this intervention, she remained with constant symptoms, significantly affecting her quality of life. She underwent cystoscopy in August 2019 showing inflammation but no mass lesions; biopsies were not performed. Urine cytology showed no malignancy, abundant inflammation, and significant Candida. CT imaging of the abdomen and pelvis was significant only for a thickened bladder wall, consistent with cystitis. Given the persistent symptoms, pyuria, and relatively negative urine cultures (Table 1), she was treated with antifungal medication for the possibility of a true candida cystitis and had a urine sample sent for AFB culture. Given no improvement, she was started on silver nitrate bladder instillations by urology for symptomatic relief. The first treatment of silver nitrate bladder instillation led to an improvement in discomfort which was regrettably short-lived.

Prior to the second planned instillation of silver nitrate, her urine AFB culture was positive for *Mycobacterium avium* complex via the nucleic acid DNA probe (AccuProbe Mycobacterium avium complex culture identification test, Hologic). In October 2019, she was started on an antimicrobial regimen of clarithromycin 500 mg BID, rifampin (RIF) 600 mg/day, and ethambutol 1200 mg/day based on susceptibilities (Table 2) and continued with the silver nitrate instillations for about a month given a little improvement after starting them. The instillations were discontinued due to intolerable bladder spasms. Unfortunately, despite antimicrobial therapy, she remained quite symptomatic and was having increasing nausea from the medications.

In January 2020, due to gastrointestinal symptoms from medications, lack of improvement in pyuria, and lack of improvement in her dysuria, the antimicrobial regimen was changed to rifampin 600 mg/day, linezolid 600 mg/day, and three-times-weekly amikacin. After the first week of this regimen, she had significant improvement in her urinary symptoms. By the end of the second week, she was completely asymptomatic. She completed three weeks of the amikacin and was then maintained on linezolid and rifampin alone. Follow-up urinalysis showed marked improvement in pyuria, and she remained asymptomatic. For the next ten months, she remained symptom-free from a urinary standpoint and tolerated the linezolid/rifampin combination with no ill effects. She completed twelve months of therapy from initiation of the amikacin with a follow-up urine analysis demonstrating no pyuria. A second urine analysis a few weeks the first was also negative. Interestingly, a second urinary AFB culture obtained prior to any antimycobacterial therapy (but after the first silver nitrate instillation) was ultimately negative.

## 3. Nontuberculous Mycobacteria: An Overview

Among numerous NTM species, MAC is the most predominant group causing disease in humans. Originally not recognized as pathogenic, in the 1950s, the isolation of acid-fast bacilli with atypical cultural characteristics was noted at Battey State hospital, Rome, Georgia USA. At this time there developed evidence of direct causation of disease [11,12]. In 1959, Runyon proposed the first classification of NTM into four groups, the first three comprising “Slowly Growing Mycobacteria” (SGM) and the fourth of “Rapidly Growing Mycobacteria” [5]. MAC is part of group III or Battey type NTM in the Runyon classification [13]. With advances in the systematic analysis we are able to identify and classify new (sub) species within MAC at the molecular level. The most common SGM is the species belonging to the *Mycobacterium avium* complex (MAC), comprising especially *Mycobacterium avium* and *Mycobacterium intracellulare*.

The incidence of MAC infections is increasing in most industrialized countries, possibly because of the increase in immunocompromised and/or older patients [1,2]. In addition, improvement in testing methods to culture and identify have spurred increases in MAC have likely led to increased diagnoses. The use of fluorescent stain and fluorescent microscope appears to be superior to Ziehl-Neelson stain for AFB smear [14]. AFB culture in liquid Mycobacteria Growth Indicator Tube (MGIT) has replaced the Lowenstein-Jensen slant in isolating NTM and DNA probes and matrix-assisted laser desorption ionization-time of flight mass spectrometry (MALDI-TOF) allows for precise identification in hours compared to the traditional biochemical and phenotypic methods that can take weeks [15,16].

## 4. Mycobacterial Genitourinary (GU) Infections: Clinical Presentation

Mycobacterial genitourinary (GU) tract infections are a not-uncommon event, and genitourinary tract tuberculosis is a common manifestation of extrapulmonary TB. In genitourinary tuberculosis, men are more frequently infected than women, and HIV-infected men are at the highest risk [17]. Initial presentation is not always associated with specific symptoms; pyuria and/or microscopic hematuria may be observed as incidental findings. Involvement of the bladder may cause symptoms of frequency, dysuria, urgency, and nocturia in approximately half of the cases; gross hematuria and low back pain develop in 1/3 of cases. Systemic symptoms (fever, weight loss) are relatively rare [18]. While there are good data on prevalence and therapeutic options for tubercular GU infections, non-tubercular (NTM) infections of the GU tract are much less common, and there is currently no standard approach to therapy.

Most cases that have been documented of genitourinary NTM showed increased prevalence in women. Infections are more common in the elderly population, although a case of genitourinary MAC associated with disseminated infection has been reported in a child as young as 14 years [19]. Genitourinary infection does not appear to be isolated to the immunocompromised, as only one infection in a transplant recipient has been documented [20]. Most patients appear immunocompetent. It is unclear if race is a factor; of the published cases, most patients are Caucasian, but lack of diagnosis and publication bias could play a role in this.

Primary complaints of UTI appear to be present in all those that have been diagnosed with MAC as the etiology of their symptoms. Duration of symptoms varies from 1 week to 3 years. Several patients appear to have been treated with antibiotics for suspected bacterial urinary tract infections in the past. Sterile pyuria in routine urine culture with dysuria appears to be a common presenting sign. Of interest is the duration of symptoms and persistence of symptoms after routine treatment with antibiotics. In our patient, she has grown multiple bacterial organisms in the urine cultures over the years including Candida species with some improvement in symptoms until the next recurrence. Other NTM (*M. abscessus*, *M. fortuitum*, *M. gordonae*) causing genitourinary infection do not have a similar presentation [21,22,23,24,25,26,27].

The most common species of rapidly growing mycobacteria of clinical relevance are *M.fortuitum, M.abscessus, M.marinum,* and *M.chelonae.* While predominantly known to cause skin and soft tissue infections, these pathogens can cause disease in other parts of the body, including the GU tract, and there are increasing published reports of these organisms leading to infection in this organ system. *M. fortuitum* has been associated with prolonged urinary tract elimination in patients with AIDS as well as hemodialysis patients [23,25]. While it is not always clear that the presence of these pathogens in a urine culture is necessarily reflective of true infection, in several cases there has been clinical evidence of true disease, and antimicrobial treatment led to culture sterilization and clinical improvement (Table 3) [20,28,29,30,31,32].

Urinary involvement with disseminated spread has been associated with underlining cell-mediated immune deficiencies or serum interferon-Y-neutralizing autoantibodies [19,28]. None of the patients appear to have any history of HIV which has been associated with disseminated MAC. One of the patients that completed treatment and had sterilization of urine was found to have a recurrence after undergoing orthotopic liver and kidney transplant and being placed on immunosuppression. It is unclear if immunosuppression definitively increases the risk of isolated GU infection secondary to MAC as noted in Obeid et al. [20].

Studies question the clinical significance of MAC in the urine as some patients are not treated with antibiotics and managed conservatively [33,34,35]. Like other potential pathogens isolated on cultures, the presence of a positive culture does not necessarily mean true infection: in one large New York hospital over a 3-year period, there were 422 HIV-negative patients with cultures positive for MAC from various body sites. Of these, 375 had clinical data available to determine true mycobacterial infection or not, of which only 119 felt to have a true infection. One patient had repeated positive urine cultures for MAC but was not categorized as having NTM disease since the patient was asymptomatic [36]. Additionally, it has been suggested that about 43% (12/28 tested) of HIV+ individuals with disseminated MAC infection will have positive urine cultures for this organism [37]. Two reported patients continued to be symptomatic, despite achieving urine sterilization, which raises the question if treatment was beneficial or necessary [31,32]. Two patients had renal involvement requiring surgical management in addition to antibiotic therapy which led to symptomatic improvement and urine sterilization [29,30]. Thus, proper source control may have provided a similar effect. Given the lack of susceptibility information or standardization of care with antibiotics and duration of treatment, it is difficult to form a firm conclusion.

## 5. Mycobacterial GU Infections: Diagnosis

General practice for patients with ongoing culture-negative urinary symptoms often involves cultures for atypical organisms (such as Chlamydia, Gonorrhoea, Mycoplasma, Ureaplasma, and mycobacterial organisms). Molecular techniques have been explored, and one study used nucleic acid probes of tissue from bladder wall biopsy of patients with interstitial cystitis to look for missed mycobacterial pathogens; of the eight tested, none were positive suggesting that this may not be a common cause of ongoing culture-negative urinary symptoms in this small study [38]. However, there are clear cases where MAC has been implicated in true GU tract infections (Table 3) with consistent symptoms, positive cultures, and clinical benefit from antimicrobial therapy. However, the optimal treatment of medications and duration is not clear at this time.

Our patient exemplifies the difficulty in making a diagnosis of NTM GU tract infection. She had ongoing symptoms for years, constant pyuria on cultures, and was treated repeatedly for small-quantity colonizing bacteria with no clinical improvement. In addition, like many patients with recurrent GU issues, she had more than one problem along the way: initially she had urinary retention, and later developed colonized/infected kidney stones. AFB culture was sent after evaluation by infectious diseases clinic and returned positive after four weeks of incubation. Prior to starting therapy, the culture was repeated, but ultimately reported as negative perhaps as a result of the intermittent intravesicular silver nitrate installations for symptomatic relief. Studies have shown silver nanoparticles (AgNPs) demonstrate antimycobacterial effect in bacterial cultures and within macrophages [39]. The use of monthly silver nitrate installations in conjunction with antibiotic management may have been the key to the eradication of the infection in this patient.

## 6. Mycobacterial GU Infections: Treatment

Treatment for genitourinary mycobacterial infection has not been standardized. Initial regimens appeared to favor isoniazid (INH), ethambutol (EMB), RIF, and streptomycin [29,30,31,32]. All except Faber et al. used three drug regimens to initiate treatment with modifications as needed for each patient, despite which urine sterilization was achieved. Recent case reports show a shift to macrolide (azithromycin or clarithromycin) based treatment with the use of Clinical and Laboratory Standards Institute (CLSI) published guidelines for drug susceptibility testing (DST) of NTM in 2011 and updated in 2018. Along with macrolides, RIF, EMB, fluoroquinolones, and aminoglycosides have been used as additional medications for three drug antibiotic therapy [20,28].

Treatment for genitourinary MAC does appear to be extrapolating from pulmonary MAC treatment which is based on macrolides combined with a rifamycin (rifampin or rifabutin) and ethambutol. The use of amikacin can be parenteral or inhaled in for severe cases of pulmonary MAC [7,40], although older case reports show streptomycin (600 mg-1g) as the most common addition in 5 out of 7 cases [28,29,30,31,32]. Moxifloxacin, bedaquiline, linezolid, and clofazimine are alternative drugs proposed mainly for the treatment of infections caused by macrolide-resistant pulmonary MAC [41].

In NTM lung disease, CLSI and the British Thoracic Society (BTS) recommend testing the isolate for clarithromycin and amikacin susceptibility prior to the initiation of therapy. It is reasonable to assume that genitourinary infections would benefit from similar testing. In cases of resistance to clarithromycin, CLSI recommends DST of moxifloxacin and linezolid, whereas BTS recommends testing a wider panel of antibiotics to guide treatment regimens [42,43,44]. Ethambutol and rifamycins, unfortunately, have no clinical breakpoints defined for treatment of MAC infections [41].

The use of these antimicrobials does require further evaluation regarding pharmacokinetics with special consideration of urine concentration achieved by each. Linezolid, clarithromycin, clofazimine, and aminoglycosides (streptomycin and amikacin) achieve the highest levels in urine as noted in Table 4. It is not certain how relevant urine antimicrobial concentrations are, as the use of oral medications with lower urinary concentration often leads to urinary sterilization.

Based on the minimal amount of published data on *Mycobacterium avium* complex genitourinary infections, optimal therapy and duration are unclear at this time. Patients have been treated with several regimens with varying duration of treatment ranging from 6 weeks to 18 months. While likely rare enough to make randomized trials of different therapeutic options impossible, this case suggests that relatively brief therapy with amikacin may be of benefit. The practice of confirming pathogen clearance with repeat sputum culture in pulmonary MAC could be transitioned to genitourinary MAC infection with the determination of duration of treatment after urine sterilization. While the ideal duration of both aminoglycoside therapy and total therapy is unclear, patient symptoms and degree of pyuria may be of benefit in measuring disease activity even in the face of negative follow-up cultures.

## 7. Conclusions

Genitourinary *Mycobacterium avium* complex infection appears to be rare. Patients commonly have symptoms with dysuria and sterile pyuria for prolonged periods prior to proper diagnosis. Concern of clinical relevance despite urine culture positivity is present although our case in addition to prior reports of similar patients confirms the need for aggressive and prolonged management to provide symptomatic relief when true infection, and not just colonization, is present. Based on data from the more-common pulmonary infections, beginning with three-drug therapy and evaluation of sensitivity to macrolides and aminoglycosides is recommended. A short course of aminoglycoside for genitourinary MAC may be beneficial in some patients. Overall duration of therapy is unclear, as literature review shows a wide range of therapy, ranging from six weeks to 18 months. Optimal medication choice and antibiotic duration may require tailoring to each individual patient.

## Figures and Tables

**Table 1:** Urine culture results 2016 to present.

Date	Leucocyte Esterase	WBC/HPF	Culture
1/9/16	Large	>100	>100K *Pseudomonas aeruginosa*, 20K MSSA
2/15/16	Small	10–20	50K MSSA
5/6/16	Large	>100	>100K MSSA
5/10/16	Large		60K MSSA
5/21/16	Large	>100	50K *Candida albicans*
6/18/16	Large	>100	>100K *Citrobacter freundii*, >100K *Enterococcus faecalis*, 10K mixed gram-positive flora
6/19/16	Large	50–100	>100K *Candida albicans*
6/28/16			40K *Candida albicans*
8/31/16	Small	10–20	>100K *Proteus mirabilis*
9/4/16	Large	Packed field	>100K *Candida albicans*
6/19/17			>100K MSSA
9/1/17	Large		>100K MSSA
9/15/17	Large		50K MSSA
9/20/17	Large	>100	40K MSSA
2/6/18	Large		5K mixed Gram-positive flora
7/16/18	Large		40K MSSA
8/21/18	Large		30K MSSA, 6K mixed Gram-positive flora
11/16/18	Moderate		>100K MSSA
1/7/19	Moderate		20K MSSA
1/19/19	Small	20–50	400 CFU yeast
3/9/19	Moderate	>100	2K yeast
8/19/19	Moderate	Packed field	4K yeast
9/5/19			*Mycobacterium avium* complex
10/15/19	Large		AFB culture negative
11/5/19	Moderate		30K *Enterococcus faecalis*, 20K yeast
12/8/19	Moderate	>100	20K normal urogenital flora
12/23/19	Large		20K yeast, 10K *Enterococcus faecalis*
1/3/20	Large	Packed field	50K yeast
9/20/20	Negative	<5	No growth
2/25/2021	Negative	<5	No growth

WBC/HPF—white blood cells/high power field, MSSA—methicillin-susceptible *Staphylococcus aureus*, AFB—acid-fast bacillus, CFU—colony forming unit.

**Table 2:** *Mycobacterium avium* complex susceptibility.

Antimicrobial	MIC	Interpretation
Amikacin IV	2	Sensitive
Amikacin—Liposomal, Inhaled	2	Sensitive
Clarithromycin	0.25	Sensitive
Linezolid	≤1	Sensitive
Moxifloxacin	0.25	Sensitive

Abbreviation: MIC, minimum inhibitory concentration.

**Table 3:** MAC case reports.

Year	Reference	Age/Sex	Race	Clinical Findings	Duration of Symptoms	Organism in Urine	Susceptibility	Treatment	Outcome
1963	Faber et al. [29]	27/F	Japanese—American	Gross hematuria. Left renal mass	1 week	Atypical AFB (Battey type)	N/A	Empirically started Streptomycin 1 g/day, INH 250 mg/day. One month in decrease streptomycin to twice a week. Total duration 4 months	Removal of left renal mass (pathology showed no AFB) after surgery pt was asymptomatic
1966	Newman, H. [30]	52/F	Caucasian	Pyuria and dysuria	10 days, Intermittent dysuria since teenager, similar symptoms after 2 years	2 cultures grew 3 atypical mycobacteria and one group 4 rapid growers	N/A	Streptomycin 1 g twice a week, INH 300 mg/day + PAS 12 g daily for 12 months, after which INH and PAS were continued for another 6 months	Symptomatic improvement. Had left nephroureterectomy which showed granulomatosis changes consistent with tuberculosis. Urine sterilization after procedure.
1973	Pergament et al. [31]	62/F	Presumed Caucasian	Frequency, urgency, nocturia, suprapubic pain and right lower quadrant pain with gross hematuria	6 months	Battey-avian complex	Sensitive: INH, RIF, EMB	INH 300 mg/day, RIF 600 mg/day, EMB 15 mg/kg/day and Streptomycin 1 g 3 times a week for total of 6 weeks	Patient continued to have symptoms but achieved urine sterilization
1986	Mikolich et al. [32]	75/M	Presumed Caucasian	Granulomatous Prostatitis, Difficulty urinating with hematuria and pyuria	1 year	MAC	Initial culture: Sensitive: PZA; Resistant: INH, EMB, streptomycin and RIF. CDC sensitivities to initial culture: Sensitive: ansamycin Resistant: Capreomycin, streptomycin, INH, PAS, RIF, EMB, kanamycin, PZA, cycloserine and ethionamide	INH 300 mg/day, RIF 600 mg/day × 4 months, EMB 1 g/day + PZA 1.5 g/day. No change, 6 weeks of ansamycin 300 mg/day, INH 300 mg/day, EMB 1 g/day + 1 g of streptomycin IM 3 times a week for 1 week then twice weekly for total of 6 weeks	No improvement with treatment. Some improvement with NSAIDs
2015	Obeid et al. [20]	61/F	Somali born	Liver cirrhosis with chronic dysuria, s/p transplant with recurrence	Unclear duration	MAI	Sensitive to Clarithromycin, Moxifloxacin, Linezolid	Azithromycin 250 mg/day, EMB 1200 mg/day, RIF 600 mg/day (substituted rifabutin), Moxifloxacin 400 mg/day—17 months total	Urine sterilization in 5 months and completed 17 months of treatment. With 1 year of therapy after first negative mycobacterial urine culture. Recurrence after 8 months of orthotopic liver and kidney transplant. Refused treatment and pt died unrelated to MAI
2018	Miyashita et al. [28]	63/F	Presumed Japanese	Disseminated MAC initially presented with fever, eruption and sterile pyuria	Unclear duration	MAC	N/A	Clarithromycin 800 mg/day, RIF 450 mg/day, EMB 750 mg/day, (streptomycin 600 mg/day × 3 days—2 months)	1 month after treatment afebrile, resolution of urinary incontinence—Regression of multiple organ involvement except splenic lesions—Over 20 months of therapy
2019	Present case	79/F	Caucasian	Recurrent symptomatic UTI	3 years	MAC	Sensitive: Amikacin, Clarithromycin, Linezolid and Moxifloxacin	Clarithromycin 500 mg po BID, RIF 600 mg po daily and EMB 1200 mg po daily changed treatment after 4 months to continue RIF 600 mg/day, linezolid 600 mg/day, and three-times-weekly amikacin for 3 weeks.	Asymptomatic after 12 months of treatment. Urine sterilization.

AFB—acid-fast Bacillus, N/A—not available, INH—isoniazid, PAS—para-aminosalicylic acid, *RIF*—rifampin, EMB—ethambutol, PZA—pyrazinamide, MAI—*Mycobacterium Avium*-*Intracellulare*, MAC—*Mycobacterium avium* complex, UTI—urinary tract infection, NSAIDs—nonsteroidal anti-inflammatory drugs.

**Table 4:** Pharmacokinetics of antimycobacterial drugs with urine concentration.

Drug	Dose	Urinary Drug Level (μg/mL)
Clarithromycin [45]	500 mg BID	~1.24
Azithromycin [46]	500 mg qd	~148
Rifampin [47]	600 mg qd	400–600
Ethambutol [48]	25 mg/kg/d	7–9
Streptomycin [49]	600 mg IM qd	174–534
Amikacin [50]	500 mg IM qd	600–832
Linezolid [51]	600 mg BID po	192–61
Moxifloxacin [52]	400 mg qd po	~137.6
Bedaquiline [53]	400 mg po qd	~4
Clofazimine [54]	100 mg 3 times/wk	156–456

## Data Availability

Not applicable.

## Abbreviations

AFB	Acid-fast bacillus
AgNPs	silver nanoparticles
AIDS	Acquired immunodeficiency syndrome
BTS	British Thoracic Society
CLSI	Clinical and Laboratory Standards Institute
DST	Drug susceptibility testing
EMB	ethambutol
GU	Genitourinary
HIV	Human immunodeficiency virus
INH	isoniazid
MAC	Mycobacterium avium complex
MAI	*Mycobacterium Avium*-*Intracellulare*
MALDI-TOF	matrix-assisted laser desorption ionization-time of flight mass spectrometry
MGIT	Mycobacteria growth indicator tube
NTM	Nontuberculous mycobacteria
RIF	Rifampin
SGM	Slowly growing mycobacteria
UTI	Urinary tract infection
